# Quantifying the Escape Mortality of Trawl Caught Antarctic Krill (*Euphausia superba*)

**DOI:** 10.1371/journal.pone.0162311

**Published:** 2016-09-13

**Authors:** Bjørn A. Krafft, Ludvig A. Krag, Arill Engås, Sigve Nordrum, Inge Bruheim, Bent Herrmann

**Affiliations:** 1 Institute of Marine Research, Bergen, Norway; 2 DTU Aqua, Technical University of Denmark, Hirtshals, Denmark; 3 Aker BioMarine AS, Oslo, Norway; 4 Rimfrost AS, Fosnavåg, Norway; 5 SINTEF Fisheries and Aquaculture, Fishing Gear Technology, Hirtshals, Denmark; University of Shiga Prefecture, JAPAN

## Abstract

Antarctic krill (*Euphausia superba*) is an abundant fishery resource, the harvest levels of which are expected to increase. However, many of the length classes of krill can escape through commonly used commercial trawl mesh sizes. A vital component of the overall management of a fishery is to estimate the total fishing mortality and quantify the mortality rate of individuals that escape from fishing gear. The methods for determining fishing mortality in krill are still poorly developed. We used a covered codend sampling technique followed by onboard observations made in holding tanks to monitor mortality rates of escaped krill. Haul duration, hydrological conditions, maximum fishing depth and catch composition all had no significant effect on mortality of krill escaping 16 mm mesh size nets, nor was any further mortality associated with the holding tank conditions. A non- parametric Kaplan-Meier analysis was used to model the relationship between mortality rates of escapees and time. There was a weak tendency, though not significant, for smaller individuals to suffer higher mortality than larger individuals. The mortality of krill escaping the trawl nets in our study was 4.4 ± 4.4%, suggesting that krill are fairly tolerant of the capture-and-escape process in trawls.

## Introduction

In a regulated catch quota system, estimating unaccounted mortality is a vital factor in the overall estimation of total fishing mortality [1,2]. Unaccounted mortality includes the deaths that occur after escaping the fishing gear, due to physiological damage, stress or trauma–factors which may also increase vulnerability to predators [3,2,4]. Antarctic krill (*Euphausia superba*, hereafter krill), are circumpolar in distribution and constitute an important fishery resource [5,6,7,8]. Krill are regarded as one of the most under-exploited fisheries in the world [9,10], with a potential harvest from the Scotia Sea and southern Drake Passage equivalent to 7% of current global marine fisheries production [11]. The distribution and level of the krill harvest is expected to expand [7], but the methods for estimating unaccounted fishing mortality in krill remain poorly understood.

Trawlers involved in the krill fishery use various trawl designs, with different mesh sizes, and estimates of the size-selectivity of various gears shows that escape occurs even from some of the smallest meshes used commercially [12]. Underwater video recordings made during commercial trawling indicate that the orientation of the animals escaping the meshes is not random; escapees usually exit the trawl head first and relatively perpendicular to the netting wall [12]. This suggests that individual krill may be able to orientate themselves optimally in relation to the trawl and that this behavior could theoretically increase the proportion escaping. Alternatively, the escape process may be more random, since a 200 m long commercial trawl provides many opportunities for krill to contact the netting during their journey to the codend and at some point individuals may meet the netting at an optimal orientation purely by chance. The estimated 50% retention body length (L50) of krill in the commonly used 16 mm mesh size was 33.91 mm [12]. Because many of the length classes of krill can escape through the commonly used mesh sizes, it is important to estimate the survival of escapees from these fishing gears to achieve responsible harvest levels and sustainable management. If the escape mortality is high, non-selective mesh sizes would be preferable.

Siegel estimated the escape mortality rate of krill at 5–25% [13], based on the assumption that the mortality rate of the individuals passing through the net meshes equals the rate of lethally damaged individuals observed in the codend of the commercial trawl. However, Broadhurst et al. [14] reported that inspection of damaged individuals from a trawl catch is a poor proxy for mortality. But if such values are correct, the total mortality caused by the commercial fishery might be considerably higher than reported catch values. More formal estimates of unaccounted fishing mortality have been difficult to obtain, often due to the complex logistics involved in survival studies (see review in [14]). Organisms escaping from fishing gear must be subsequently and gently recaptured. A common approach used to collect escapees from trawls involves attaching fine meshed bags or covers to or around the trawl body, or more often to the codend [15,16,17]. The collected escapees are then gently transferred to holding tanks or other enclosures in the field, which mimick natural conditions, to assess any delayed mortality [18,19].

Studies of survival of escapees have been carried out for many different species worldwide (reviews in [20,14]) and show great variability in species survival, reflecting differences in species robustness and their ability to withstand physical stress and fatigue. Crustaceans have a higher chance of survival compared to fish since their durable exoskeletons provide increased protection against abrasion and compression [17,21,22,23].

Development and initial testing of a trawl based sampling technique to monitor mortality rates of escaped krill employing a covered codend technique followed by onboard observations in holding tanks have been published [24]. The results suggest that krill are probably fairly tolerant to the capture-and-escape process, which is consistent with studies involving other crustaceans [25,26,23]. The results also suggest that krill with smaller body lengths suffered higher mortality. However, the large variation in the mortality rate observed between relatively few replicates indicates inadequate holding tank conditions. However, based on the accumulated experience from these trials, Krafft and Krag [24] made several recommendations to increase the accuracy of the estimated escape mortality for potential future studies.

This study set out to quantify the escape mortality of trawl caught krill, following the study design and recommendations for methodological improvements given in Krafft and Krag (24): i) increased number of replicates; ii) establishment of adequate experimental control groups; and iii) optimized holding facilities to mimic natural conditions as closely as possible. In addition, we provide a formal statistical approach to investigate mortality rates of escapees against time, applying a non-parametric Kaplan Meier (KM) model [27] to the data.

## Materials and Methods

### Ethical statement

This study did not involve endangered or protected species. Experimental fishing was conducted on board a Norwegian commercial trawler. No permit was required to conduct the study on invertebrates. Field permit was granted by CCAMLR (Commission for the Conservation of Antarctic Marine Living Resources).

### Data collection

**Fig 1:**
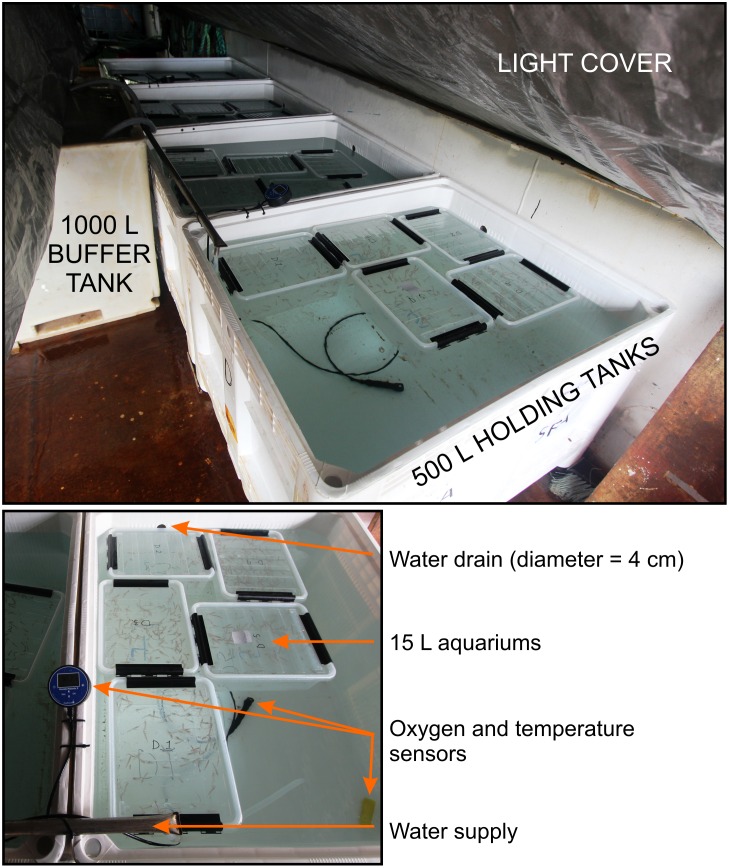
Experimental holding tank set-up with krill control groups and trawl caught escapees to monitor escape mortality.

This study was carried out on commercial fishing grounds off the coast of the South Orkney Islands (60°35′S, 45°30′W) [28] during February 2015. The vessel used was the FV *Juvel* (Olympic AS) a Norwegian, 99.5 m, 6000 kw/8158 hp (main engine) commercial ramp trawler. Trawls were performed on acoustic registrations, using Simrad EK60 General Purpose Transceivers connected to hull mounted ES60 transducers. The trawl used for the experiment had a 6 × 6 m mouth opening, fitted with a 7 mm cover for the 16 mm codend. The trawl body and cover were supported by an outer 200 mm protection net (see further details regarding the trawl design below). Krill were captured to establish a control group for the survival experiment by closing the cover and keeping the inside codend open. An initial haul provided 2.0 kg krill which were used to establish a control group for the survival experiment. These krill were distributed between eight 15 L aquariums (n = 42–193 in each/aquarium). Two aquariums were placed in each of the four 500 L holding tanks (Fig 1). During the first 24 hours, the krill in the aquariums were regularly checked for visible signs of abnormal swimming activities, discoloration due to punctured haemocoel or other potential physical damage. A total of 24 hrs after this haul was taken on board, the control group was considered established since no individuals had to be removed from any of the eight control aquariums (Table 1). With the control established, the covered-codend experiment [21] proceeded to collect replicates to monitor the survival of escapees.

**Table 1:** Summary of mortality inspections made for control groups. X: no inspection made.

Inspection time (day:hour:min)	No. dead Box A1	No. dead Box A2	No. dead Box B1	No. dead Box B2	No. dead Box C1	No. dead Box C2	No. dead Box D1	No. dead Box D2	Total
06:12:05 (on deck)	0	0	0	0	0	0	0	0	0
06:13:12	0	0	0	0	0	0	0	0	0
07:12:30	0	0	0	0	0	0	0	0	0
07:19:18	0	0	0	0	0	0	0	0	0
08:14:30	0	0	0	0	0	0	1	0	1
09:00:25	0	0	0	0	0	0	1	0	1
09:10:00	0	0	0	0	0	0	0	0	0
09:22:00	0	0	0	0	0	0	0	0	0
10:10:00	0	0	0	0	0	0	1	0	0
10:22:00	0	0	0	0	0	0	1	0	0
11:08:30	0	0	0	0	0	0	0	0	0
11:22:00	0	0	0	0	0	1	0	0	1
12:13:00	0	0	0	0	0	0	0	0	0
12:23:00	0	0	0	0	0	0	0	0	0
13:12:53	0	0	0	0	0	0	0	0	0
13:22:00	0	0	0	0	0	0	0	0	0
14:13:00	0	0	0	0	0	0	0	0	0
14:17:20	0	0	X	X	0	0	0	0	0
14:18:30	0	1	X	X	0	0	0	0	1
14:20:05	0	0	X	X	0	0	0	0	0
14:22:04	0	0	X	X	0	0	0	0	0
15:01:50	0	0	X	X	0	0	0	0	0
15:12:19	0	0	0	0	0	0	0	0	0
**Total no. live krill**	**73**	**68**	**88**	**65**	**61**	**45**	**117**	**84**	**601**
**Total no. dead krill**	**0**	**1**	**0**	**0**	**0**	**1**	**4**	**0**	**6**

**Fig 2:**
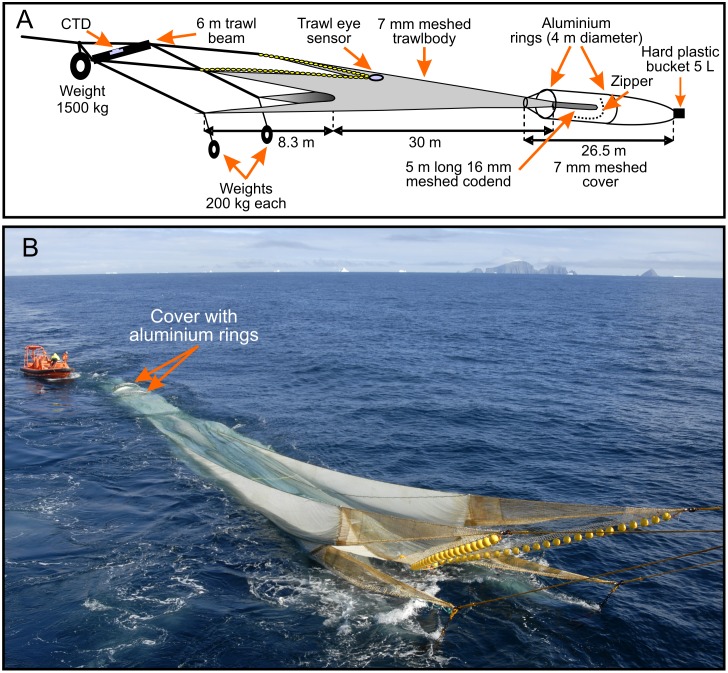
Covered codend sampling system used to collect krill trawl escapees (A (see also Fig 1 in Krafft and Krag [24] and B).

**Fig 3:**
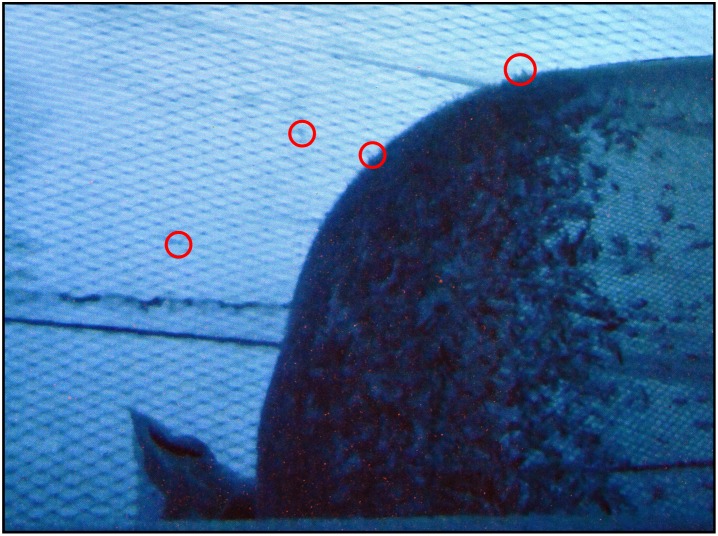
Image captured inside the cover facing the codend (located at the right side of the picture) during fishing, using underwater video, Red circles indicate krill penetrating 16 mm meshes in the codend and escapees within the cover. The cover mesh was 7 mm supported by a 200 mm protection net.

The trawl had a 5 m long codend with 16 mm netting (standard commercial mesh size) and a 26.5 m long cover net (7 mm stretched mesh) was added to collect any krill escaping (Fig 2). The cover net was stretched using a hoop cover design (two aluminum rings, of 4 m diameter) to prevent masking the codend. We used underwater cameras mounted inside the cover, facing the codend, to inspect the system (GoPro Hero 3 cameras in aluminum housings (IQsub, 300 m water resistant)) (see Fig 3).

We suspected that larger catches of escaped krill in the cover might impact the animals’ metabolism due to reductions in oxygen concentration. In addition, their increased exposure to mechanical damage due to denser packing and prolonged handling time on deck before transfer to the holding facilities might contribute to further increased mortality. Smaller catches were therefore preferred and we took steps to try to limit catch size. Krill that had escaped from the codend were collected from the rear part of the cover using a 5 L hard plastic bucket with holes covered by 500 μm mesh netting to enable sieving of seawater during towing and landing, reduced krill mobility and layered packing of krill as individuals enter the bucket. The plastic bucket was attached to a hard nylon column and the rear cover rigged with a quick release system to enable fast transfer of the krill to the holding facility.

Hydrographic data were acquired using a mini CTD (Star–Oddi) mounted to the trawl beam, logging at 10-second intervals (Table 2), and a trawl eye sensor (type A1, www.marport.com) attached to the headline gave depth and temperature information during fishing operations. The trawl was towed at commercial speeds of about 2.0–2.5 knots.

**Table 2:** Operational conditions and survival probability 60 hours (P60) after trawl arrived on deck for codend mesh escapement hauls.

Haul no.	Max. depth (m)	Haul duration (min.)	Min. temperature (°C)	Temperature surface (°C)	Salinity Mean ± SD	Cover catch (kg)	Codend catch (kg)	P60
1	152	36	-1.4	1.2	33.4 ± 0.3	0.06	0	0.99
2	165	34	-1.2	0.6	33.3 ± 0.1	0.5	10	1.00
3	185	46	-1.2	0.8	33.3 ± 0.2	0.05	1	0.98
4	126	42	-1.3	0.9	33.0 ± 2.7	6	58	0.98
5	191	30	-1.2	0.7	33.2 ± 0.3	7	50	0.94
6	93	36	-1.1	0.6	31.3 ± 5.6	0.5	9	0.98
7	111	53	-1.1	-1.1	33.1 ± 3.0	0.25	15	0.88
8	22	30	0.0	0.1	33.1 ± 0.1	15	84	0.90

After each haul the entire towing rig with opened codend and cover was cleaned by dragging it on the surface for 10–15 min and then hung and flushed on deck to wash out any krill remaining from the previous haul. Of a total of 17 hauls, eight were successful in catching krill in the cover (shown as hauls 1–8; Table 2). The hauls were performed day and night, to reflect commercial fishing practice.

### Experimental conditions

Surface seawater was pumped directly on board into a 1000 L insulated buffer tank via the vessels saltwater intake system. Two pumps (Fountain Pumps, Allegro) delivered 440 L water/hr into each of the four 500 L holding tanks used for this experiment (Fig 1). The buffer tank system was chosen to reduce the possibility of ambient oxygen oversaturation in the turbulent water delivered from the vessel’s large internal pump system. The high level of water exchange was chosen to most closely resemble the natural temperature conditions. The four 500 L holding tanks were fitted with a light cover (tarpaulin), hydrological conditions were monitored continuously using oxygen sensors (Oxyguard Handy Polaris 2) and mini CTDs (Star–Oddi) recorded temperature and salinity every 10 sec (Table 3). Groups of krill and krill replicates were held and separated using 15 L transparent plastic aquariums and the krill were then placed into the four 500 L holding tanks. The aquariums were perforated with 3 mm diameter holes, 320 on the side walls and 100 in the lid, to ensure sufficient exchange of water. The perforated 15 L aquariums had the advantage of reducing vessel induced movement of the individuals held in the aquariums while in the 500 L holding tanks, as well as separating the different experimental groups. The entire experimental set-up, including sensors and circulating water in all of the tanks (1000 L, 500 L and 15L), was switched on 48 hours prior to the first arrival of control groups of krill to ensure that all components were functioning properly.

**Table 3:** Holding conditions during entire monitoring period 06:12:05–15:12:19 (day:hour:min).

Holding conditions	Mean ± SD
Water temp (°C)	1.0 ± 0.8
Salinity	31.9 ± 0.3
Oxygen mg/L	11.2 ± 0.3
Oxygen Sat. (%)	100.1 ± 2.1

When a trawl was landed on deck, a sample of krill was promptly poured from the 5 L hard plastic bucket into one of the 15 L aquariums filled with surface seawater. Because the krill used in the experiment were mostly from the top layer of the krill accumulated in the bucket, they probably represented individuals from the later stages of the selection process. The individually marked closed plastic aquariums representing a particular haul were then submerged into one of the four 500 L holding tanks and inspected at regular intervals to assess krill mortality. Dead individuals were removed from the aquariums, counted and measured. All length measurements in this study were made from the anterior margin of the eye to the tip of the telson, excluding the setae (±1 mm), according to Marr [29].

### Estimation of time-dependent mortality

To investigate the time-dependent probability of mortality, we fitted a non- parametric KM curve [27] to the data for individual hauls. The KM curve provides an estimate of the proportion of individuals surviving against time. The zero point for the time parameter in the analysis was set as the time when the gear arrived on deck. The survival analysis was carried out using the statistical software tool R (version 2.15.2; www.r-project.org) using the survival package with the function survfit for estimating the KM curves. In addition to the KM curve for individual hauls, we also fitted a KM curve for the survival data, pooled over all hauls of krill escaping from the codend mesh.

A KM curve was also fitted to the survival data from the control groups.

### Investigation of parameters potentially affecting the survival probability

To investigate the potential effect of different operational parameters on the survival probability of krill in the codend mesh escapement trials we investigated the dependency of survival rate after 60 hours (P60) for individual hauls (obtained from the individual KM curves) against the values of six operation parameters: haul duration, sea temperature, maximum fishing depth, cover catch weight, codend catch weight and seawater salinity. This was investigated by testing individual single parameter linear models for the effect of each of these parameters on P60. This analysis was conducted using the lm function in the software tool R. If any of the parameters were found to be significant (p-value < 0.05) models considering multiple parameters simultaneously were also tested.

### Estimation of the size-dependent survival probability

To investigate the potential effect of krill size on their survival probability, the krill that had escaped from the codend mesh and those in the control experiment were sorted into 1 mm size groups. The number of krill alive and dead at the end of the experiment were counted separately for the mesh escapement trials and the control trial. This provided an experimental survival rate for each length group. These data had the same structure as the codend size selectivity data [21] and the same methods that were applied to model the flexible size-selection curves could therefore be applied to the model size-dependent krill survival probability. For this analysis, we applied a flexible survival probability model *s(l)* of the form:

$$s\left(l,v\right)=\frac{exp\left(f\left(l,v\right)\right)}{1+exp\left(f\left(l,v\right)\right)}$$ (1)

where *f* is a polynomial of order *m* with the coefficients *v*_*0*_ to *v*_*m*_. We applied Eq (1) with *f* of the following form:

$$f\left(l,v\right)=\Sigma_{i=0}^{m}v_{i}\times {\left(\frac{l}{100.0}\right)}^{i}$$ (2)

where we considered the orders *m ≤ 4*. Leaving out one or more of the parameters *v*_*0*_ to *v*_*4*_ led to 31 additional models that needed to be considered as potential models for the size-dependent survival probability of krill. Estimation of the average survival probability between hauls involves pooling data from the different hauls. We used a double bootstrapping technique that accounts for both within- and between-haul variation in the survival probability. For each case analyzed, 1000 bootstrap repetitions were conducted to estimate the Efron percentile 95% confidence limits [30, 31]. Because this technique is similar to the one applied by Herrmann et al. [32], it is not described further here. We tested different parametric models for *s(l*,***v****)*, where ***v*** is a vector consisting of the parameters of the model. The purpose of the analysis is to estimate the values of the parameter ***v*** that give the most likely observed experimental data, averaged over hauls, assuming that the model is able to describe the data sufficiently well. Thus, function Eq (3) was minimized, which is equivalent to maximizing the likelihood for the observed data:

$$-\sum_{j}\sum_{l}\left\{ns_{jl}\times ln\left(s\left(l,v\right)\right)+nd_{jl}\times ln\left(1.0-s\left(l,v\right)\right)\right\}$$ (3)

where the summations are over hauls *j* and length classes *l*, and where *ns*_*jl*_ and *nd*_*jl*_ are the number of surviving and dead krill respectively.

We evaluated the ability of the model to describe the data sufficiently well based on (3) based on calculation of the corresponding p-value, which expresses the likelihood of obtaining at least as big a discrepancy between the fitted model and the observed experimental data by chance. Therefore, for the fitted model to be a candidate to model the size-dependent survival data, this p-value should not be below 0.05. Model deviance versus degree of freedom can also be applied in the model evaluation [21]. Selection of the best model among those with acceptable p-values is based on comparing the AIC values for the models. The selected model is the one with the lowest AIC value [33]. If the model with the lowest AIC value does not produce an acceptable p-value, it could be due to the model’s inability to describe the length-based structure of the data or to over-dispersion in the data. Residual plots can be used to discriminate between over-dispersion and structural problems in a model’s ability to describe experimental data [21,34].

The analysis was conducted using the software tool SELNET (Herrmann et al., 2012).

Estimating the uncertainty of the size-dependent survival probability, we took the uncertainty related to model selection [35] into account by incorporating automatic model selection into each of the bootstrap iterations carried out in the estimation procedure for estimating the uncertainty in the survival probability.

## Results

### Data collection/holding conditions

The duration of experimental trawl hauls varied from 30–53 minutes, with maximum hauling depth ranging between 22–191 m (Table 2). Catch weight of krill varied from 0–84 kg in the 16 mm codend and 0.06–15 kg in the 7 mm trawl cover. Small differences between hauling and holding hydrological conditions were recorded (Tables 2 and 3). Minimum water temperature and surface temperature during hauls were more variable than surface temperature during hauling and the temperature during the entire holding period. The mean salinity levels were slightly higher for some of the hauls, compared with the mean salinity levels measured over the entire holding period. Oxygen concentrations were high, and the holding conditions were stable and similar to natural surface conditions throughout the observation period.

### Estimation of the time-dependent survival probability

**Fig 4:**
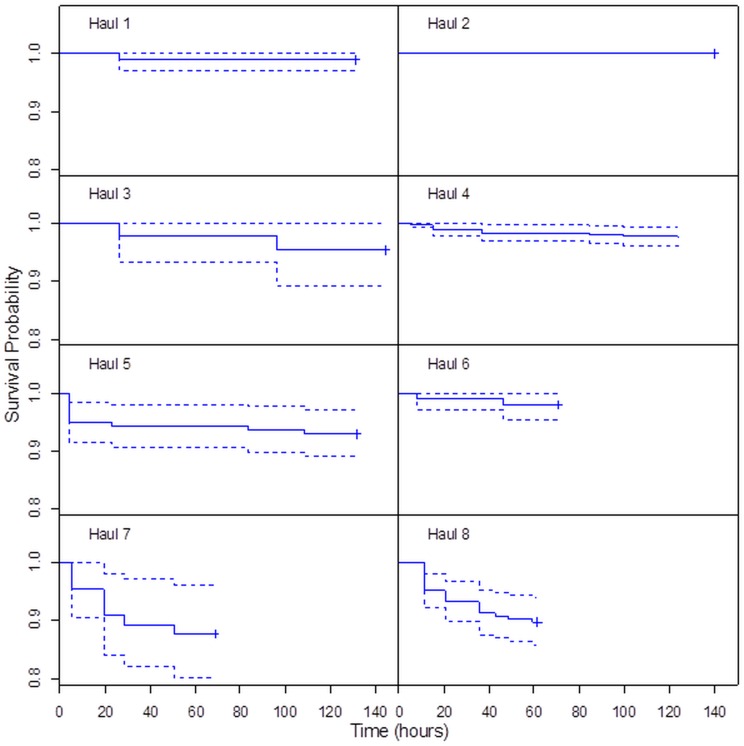
Kaplan-Meier survival probability curves for individual codend escapement hauls. Dashed lines represent 95% confidence bands. Time on x-axis is given in hours from arrival of the catch on deck.

The survival probability 60 hours (P60) after the trawl arrived on deck for codend mesh escapement hauls ranged between hauls from 0.88 to full survival; the average was 0.96 ± 0.04 (Tables 2 and 4, Fig 4). This equals a between-haul escape mortality variation ranging from 0–12%, averaging 4.4 ± 4.4%.

**Table 4:** Summary of mortality inspections made for experimental groups of escapees: T: terminated.

Inspection time (day:hour:min)	Haul no. 1 (On deck 07:12:07) No. dead	Haul no. 2 (On deck 07:17:29) No. dead	Haul no. 3 (On deck 07:21:46) No. dead	Haul no. 4 (On deck 08:09:32) No. dead	Haul no. 5 (On deck 08:10:40) No. dead	Haul no. 6 (On deck 12:14:45) No. dead	Haul no. 7 (On deck 12:17:15) No. dead	Haul no. 8 (On deck 13:01:13) No. dead
07:13:30	0							
07:19:18	0	0						
08:14:30	1	0	0	1	8			
09:00:25	0	0	1	3	0			
09:10:00	0	0	0	0	1			
09:22:00	0	0	0	2	0			
10:10:00	0	0	0	0	0			
10:22:00	0	0	0	0	0			
11:08:30	0	0	0	0	0			
11:22:00	0	0	1	1	1			
12:13:00	0	0	0	1	0			
12:23:00	0	0	0	1	1	1	3	
13:12:53	T	T	0	1	0	0	3	9
13:22:00			0	T	0	0	1	4
14:13:00			T		T	1	0	4
14:17:20						0	0	0
14:18:30						0	0	0
14:20:05						0	1	1
14:22:04						0	0	0
15:01:50						0	0	1
15:12:19						0	0	1
15:13:26						0	0	0
15:13:57						T	0	0

### Investigation of parameters potentially affecting the survival probability

**Fig 5:**
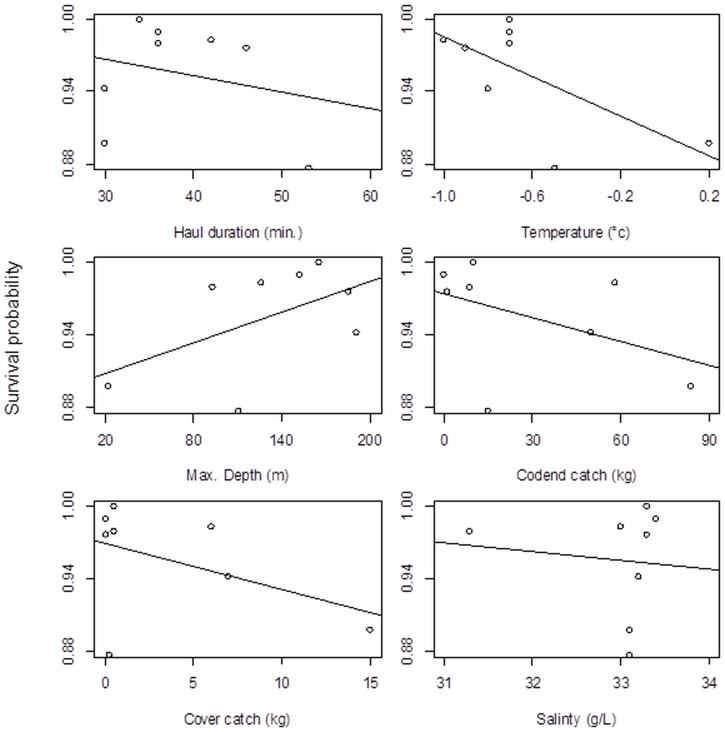
Survival probability in individual hauls 60 hours after the catch arrived on deck against different operational parameters: haul duration, sea temperature, max. fishing depth, codend catch weight, cover catch weight, seawater salinity. The lines in the plots represent the fit of the individual single parameter models.

**Fig 6:**
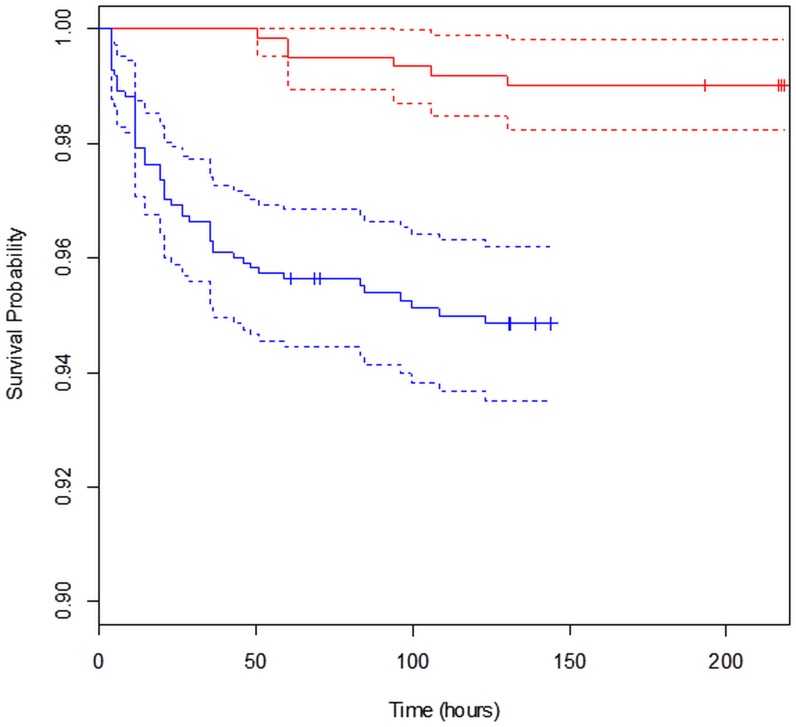
Kaplan-Meier survival probability curves for pooled hauls: codend escapement trials (blue), control experiment (red). Dashed lines represent 95% confidence limits. Time is given in hours from when the catch arrived on deck.

There were no significant effects on survival probability of individual hauls versus different operational parameters: haul duration, sea temperature, maximum fishing depth, codend catch weight, cover catch weight or seawater salinity (Table 5, Fig 5). Pooled KM survival probability curves for the codend escapement trial and control experiment show that the small mortality observed in the control groups, which includes potential mortality induced by the holding conditions, also infuenced the observed escape mortality (Fig 6). We assumed natural mortality rates to be the same between controls and experimental groups.

**Table 5:** Summary for linear models for effect on 60 hours survival rate.

Model	Intercept value	p-value for intercept	Explanatory parameter	Value for Explanatory parameter	p-value for explanatory parameter	R^2^-value
P60~Intercept + Haul duration	1.00827	2.61e-05	Haul duration	-0.00137	0.56	0.0588
P60~Intercept + Temperature	0.90311	5.03e-08	Temperature	-0.08234	0.07	0.4445
P60~Intercept + Max. depth	0.89963	5.07e-07	Max. depth	0.00043	0.18	0.2744
P60~Intercept + Cover catch	0.96979	4.08e-09	Cover catch	-0.00387	0.26	0.2074
P60~Intercept + Codend catch	0.97401	8.28e-09	Codend catch	-0.00065	0.27	0.1983
P60~Intercept + Salinity	1.19789	0.23	Salinity	-0.00735	0.89	0.0121

### Estimation of the size-dependent survival probability

**Fig 7:**
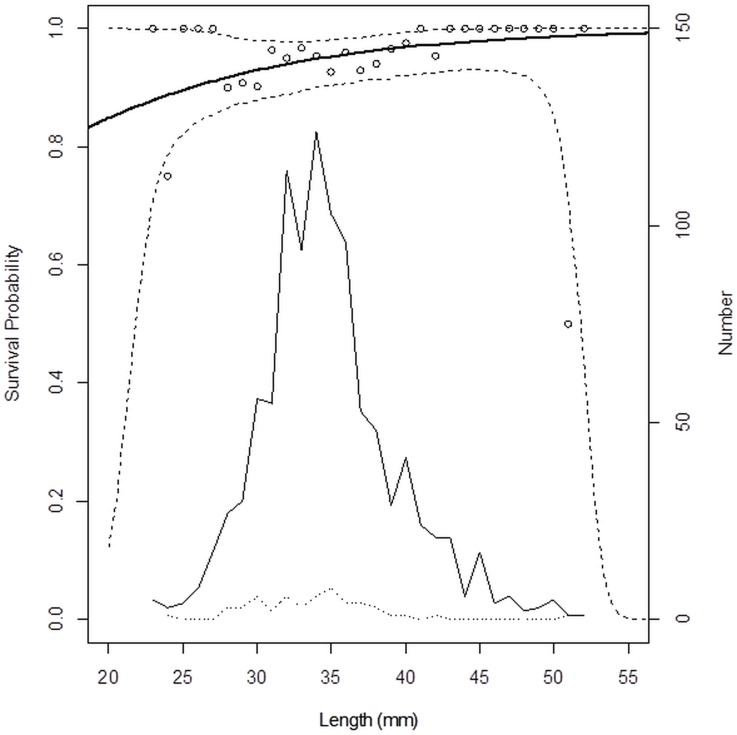
Length-dependent survival probability pooled over hauls. Circles represent experimentally observed survival probabilities. Thick solid line represents the modelled length-dependent survival rate at the end of the observation period. Dashed lines represent 95% confidence limits for the survival probability. Thin solid line shows the number of surviving krill of different sizes. Dotted line shows the number of dead krill of different sizes.

**Fig 8:**
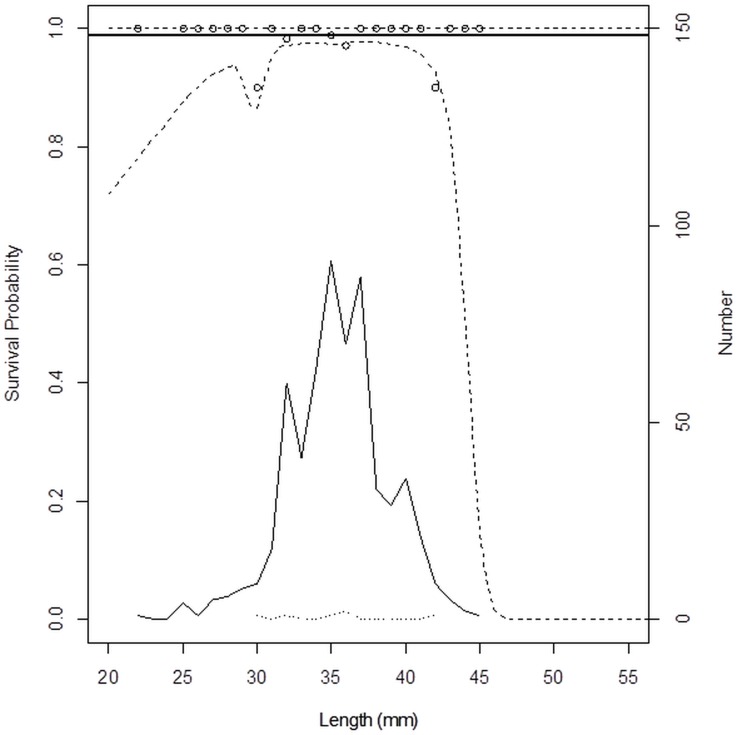
Length-dependent survival probability in control groups. Circles represent experimentally observed survival probability. Solid thick line represents the modelled length-dependent survival rate at the end of observation period. Dashed lines represent 95% confidence limits for the survival probability. Thin solid line shows the number of surviving krill of each length. Dotted line shows the number of dead krill of different sizes.

The model in Fig 7 produced a p-value at 0.70, indicating that it is likely that the discrepancies observed between data points and the model are coincidental. The model therefore describes the experimental data sufficiently well. This model has an AIC value of 422.39, while a model without the length dependency has an AIC value that is 1.58 higher (423.97). Based on this difference in AIC values, length dependency in survival probability is supported. The control groups display a linear horizontal model in this regard, indicating no length dependent mortality (Fig 8, Table 1).

## Discussion

This study set out to quantify the escape mortality of trawl caught krill. Compared to a former study [24], the current experiment involved collecting a much larger data set, it also included the establishment of adequate experimental control groups and it used improved holding facilities as similar to natural conditions as possible. Based on all the experimental improvements it was, in contrary to Krafft and Krag [24], possible to apply a formal statistical approach to investigate mortality rates of escapees against time, by using a non-parametric Kaplan Meier model. The current study thus represent significant improvement both regarding experimental design, sampling effort and on analysis of collected data and thereby provides significant progress regarding quantifying escape mortality of krill.

All eight successful experimental hauls, in which krill escaping the trawl were collected in the trawl cover and monitored on board for post-escape mortality, displayed similar mortality patterns. The highest mortality rates were observed during the first 24 hours, followed by a flattening of the survival curve (Fig 4). Our results show that the survival probability of a krill escaping the commercial trawl netting 60 hours (P60) after the trawls arrived on deck was 96%. Taking the modest between-haul variations into account, the mortality of krill escaping the codend in our study was 4.4 ± 4.4%. This clearly shows that krill are fairly tolerant of the capture-and-escape process. It also agrees with the expected escape mortality rates discussed in [24] and is consistent with studies involving other crustaceans, which also showed low mortality rates [25,26,23].

Post-escape conditions in commercial trawling situations differ from those pertaining during this experiment. Krill escaping during commercial harvests are released directly into the sea outside of the trawl body, while escapees collected with a cover face additional physical stress and environmental change during retrieval and transfer to a holding tank. We took great care during the experiment to reduce the degree of exposure to such stresses to a minimum, so as to increase the chance of isolating and studying the effect of escape on mortality. The success of this care was evident in that the variation in observed escape mortality between replicates was unaffected by any of the fixed effects. Mortality was unaffected by haul duration, exposure to different hydrological conditions, maximum fishing depth or catch composition, nor were there any negative effects associated with holding conditions. Nevertheless, other factors could be involved, such as the actual time that krill enter the trawl in relation to total hauling time. Also the critical process of hauling the trawl from the surface to the slip and up onto the deck, which was done as quickly as possible, exposed the krill to the air and possibly increased physical wear caused by the extra gravitation when out of the water. These stresses were difficult to standardize and may cause some between-haul variation in mortality rates. All things considered, our results probably represent maximum estimates for the mortality of krill escaping trawl nets.

Conventional commercial krill trawls may differ in design and operational conditions. Some are towed for up to an hour and the catch landed on deck may reach ten tonnes [36]. Other trawls may be emptied at the sea surface using a pump system, while a more recently developed “eco-harvesting technology” (patent WO2005004593), brings krill continuously to the production deck of the vessel from a submerged trawl through a hose attached to the codend. The effect on escape probabilities of various gear technologies and their mode of use (*e*.*g*. towing speed), probably differ. In general, larger catches probably reduce escape probability due to denser packing of individual krill, preventing them from orienting their bodies so as to enable penetration of the net mesh. However, the fact that escapement of krill can be significant during commercial fishing is demonstrated by comparing the population structure for the krill sampled during the experiment (Fig 7) with the expected codend mesh size selection for codends applied in the fishery. For the commonly applied codend mesh size of 16 mm; Krag et al. [12] estimated that all krill <39 mm body length have a certain probability to escape the meshes *e*.*g*.: 95% of krill less than 26 mm body length can escape and 50% of krill at ~34 mm length would be retained. If we assume that the population structure sampled during our experiment is representative for commercial fishing, then a considerable fraction of the krill entering the gear could escape the trawl gear and this demonstrates the importance of knowledge about the escape mortality in a management context.

We found indications that krill size influences survival probability, though not significantly, with smaller body sizes suffering higher mortality. It is worth noting that no such influence was found in the control groups. Krafft and Krag [24] found that small body length predicted higher mortality in their study, and speculated whether this was because the exoskeletons of smaller krill tend to be softer than those of larger krill, making them more vulnerable. A number of studies of fish demonstrate negative correlations between length and skin injury or mortality post-escape [18,37,38,39,19,40]. Such relationships might be related to size-dependent swimming ability and the possibility that larger fish make sustained escape attempts to avoid stressors such as netting panels and other parts of the towed gear that could increase physiological damage.

Animals have different tolerances for injury and it is important to understand the time requirements for this kind of holding experiment [14]. Wassenberg and Hill [41] maintained a large array of fishes and invertebrates with injuries from trawl nets for one week in laboratory tanks to understand the effects over time. They concluded that holding for four days was adequate to show permanent effects for most fishes and invertebrates. In our study, the duration of trials between hauls varied from 2.5 days to almost 6 days. This between-haul variation in monitoring time was due to the available ship time. In any case, the escape mortality signatures from the KM plots display similar survival curves with the highest mortality rates during the first 24 hours (Fig 4), indicating that the duration of our study trials was adequate for a representative description of post-escape mortality for this particular species.

Post-escape mortality studies quantify delayed mortality rates, often determined after several days. Such values do not therefore provide any information regarding conditions such as ambient stress levels that a single escapee may experience after a successful escape from the trawl. Further work on potential post-escape vulnerability to predators is still required to fully understand the effect of unaccounted fishery mortality [3,2,4]. Any possible increased predation on escaped krill could not be investigated or verified using our study design. Future studies could investigate potential post-escape vulnerability to predators in the field by measuring stress levels in the post-escape process using *e*.*g*. portable blood physiology point-of-care devices (*e*.*g*. [42]). Also the potential for sex-dependant mortality, due to sexual dimorphism, in the escape process may have an impact to the harvested stock; however this was not in focus during our study.

We observed low mortality of krill captured by a trawl and then penetrating the mesh, being transported on board and studied in holding tanks over a sustained period. The control group, which were exposed to the same stresses described above except that they did not escape a mesh, suffered almost no mortality. This shows that we succeeded in providing stable, high quality holding conditions throughout the study. The effect of escape is therefore shown by the difference in mortality between the control and experimental groups, even though the control represented only a single haul. We found low between-haul mortality variations in the escape experiment hauls, and some of this variation could be explained by stresses induced post-heaving and between holding conditions. A comparison of mortality between the control and experimental groups should ideally include several control hauls to determine whether any between-haul variations exist. We conclude that krill are fairly tolerant to the capture-and-escape process. This knowledge is valuable for the adoption of gear based management measures and for future fishing gear development to reduce escapement and unaccounted mortality, which in turn will also increase the long term economic profitability of the fishery.

## Supporting Information

S1 File S1_Aker-confirmation. (PDF)

S2 File S2_Rimfrost_confirmation. (PDF)

S3 File S3_Sintef_confirmation. (PDF)
