# Supplementary material for: Quantifying the Escape Mortality of Trawl Caught Antarctic Krill (Euphausia superba)
Source: PLoS One. 2016 Sep 13;11(9):e0162311. doi: 10.1371/journal.pone.0162311 (PMC5021277; doi:10.1371/journal.pone.0162311)
Supplement: S1 File — (PDF) [file pone.0162311.s001.pdf]

PONE-D-16-16171
Quantifying the escape mortality of trawl caught Antarctic krill (*Euphausia superba*)

This manuscript represents a part of the project "Understanding and predicting size selectivity and escape mortality in commercial zooplankton fisheries: case study on Antarctic krill" financed by the Norwegian Research Council (NRC) project no. 243619. This is a collaboration between actors from the Institute of Marine Research (project owner), DTU Aqua, Sintef Fisheries and Aquaculture (SFH), Rimfrost AS and Aker BioMarine AS. The commercial fishing companies Rimfrost AS and Aker BioMarine Antarctic AS provided ship time for conducting the fieldwork. My contribution involves design of the study, facilitating the fieldwork, decision to publish and preparation of the manuscript. I require no salary from the NRC funding for salary expenses to cover time allocated to conduct this work.

The academic Editor of PLOS ONE has requested a confirmation from Aker BioMarine Antarctic AS (AKBM) that the current manuscript and the work represented in it do not involve any Competing Interests or Financial disclosures. I hereby on behalf of AKBM also declare that the current work do not involve any competing interests or involve any financial disclosure. Therefore this does not alter our adherence to PLOS ONE policies on sharing data and materials.

Yours sincerely,

For Aker BioMarine Antarctic AS

EVP Webjørn Eikrem
on behalf of EVP Sigve Nordrum
